# Effect of probiotic supplementation on lipoprotein-associated phospholipase A2 in type 2 diabetic patients: a randomized double blind clinical controlled trial

**DOI:** 10.1186/s12986-023-00778-5

**Published:** 2024-01-02

**Authors:** Salman Jaff, Mohammed Gubari, Sakineh Shab-Bidar, Kurosh Djafarian

**Affiliations:** 1 Department of Clinical Nutrition, School of Nutritional Sciences and Dietetics, Tehran University of Medical Sciences, Tehran, Iran; 2 Department of community and family Medicine, School of Medicine, University of Sulaimani, Sulaymaniyah, Iraq; 3 Department of Community Nutrition, School of Nutritional Sciences and Dietetics, Tehran University of Medical Sciences, Tehran, Iran; 4 Neuroscience Institute, Sports Medicine Research Center, Tehran University of Medical Sciences, Tehran, Iran; 5 Diabetes and Endocrine Center, Sulaymaniyah, Iraq

**Keywords:** Lipoprotein-associated phospholipase A2, Type 2 Diabetes, Probiotics

## Abstract

**Background:**

It has been recently reported that lipoprotein-associated phospholipase A2 (Lp-PLA2) may predict the risk of cardiovascular disease. The effect of multi-strain probiotics on Lp-PLA2 in patients with type 2 diabetes is still not clear.

**Aims:**

This study aimed to determine the effect of multi-strain probiotic supplementation on lipoprotein-associated phospholipase A2, and glycemic status, lipid profile, and body composition in patients with type 2 diabetes.

**Methods:**

In this randomized double-blind placebo-controlled clinical trial, 68 participants with type 2 diabetes, in the age group of 50–65 years, were recruited and randomly allocated to take either probiotic (n = 34) or placebo (n = 34) for 12 weeks. The primary outcome was lipoprotein-associated phospholipase A2, and secondary outcomes were glycemic parameters, lipid profile, anthropometric characters, and body composition (fat mass and fat-free mass).

**Results:**

There was a significant reduction in serum lipoprotein-associated phospholipase A2, in the probiotic group, it dropped by 6.4 units at the end of the study (*p* < 0.001) compared to the placebo group. Probiotic supplementation also resulted in a significant improvement in the hemoglobin A1c and high-density lipoprotein cholesterol 1.5% (*p* < 0.001) and 6 mg/dl (*p 0.005*), respectively. There were no significant changes in other outcomes.

**Conclusion:**

Probiotic supplementation was beneficial for reducing Lp-PLA2 and hemoglobin-A1c and improving high-density lipoprotein cholesterol, which may suggest an improvement in the prognosis in patients with type 2 diabetes.

## Introduction

Diabetes is a prominent cause of death in many countries and is a key health problem both regionally and internationally [1]. It was estimated by the International Diabetes Federation that prevalence of type 2 diabetes (T2D) from 8.5 to 13.9%, and that this number could rise to 700 million (10.9%) by 2045 [2]. The occurrence of diabetes increased from 5% in 1978 to 19.7% in 2012 in Iraq, with some regions having a dysglycemia prevalence of 48.8% [3]. Type 2 diabetic patients show higher levels and prevalence of cardiovascular incidents [4]. For the pathogenesis of both heart disease and diabetes, inflammatory processes have been progressively more recognized as a crucial step which may provide a biological link between the two diseases [5].

Lipoprotein-associated phospholipase A_2_ (Lp-PLA_2_) is secreted by macrophages and is one of the newly recognized inflammatory biomarkers. Lp-PLA_2_ is an enzyme that belongs to the phospholipase A2 family that may impact plaque rupture and atherogenesis [6–8]. Both Lp-PLA_2_ activity and mass were higher in type 2 diabetic patients than in non-diabetic individuals. An increase in the levels of this enzyme was linked to a higher risk of coronary heart disease amongt diabetic patients [9]. Therefore, the enzyme could be considered as a possible therapeutic aim to lower atherosclerotic risk and the development of cardiometabolic impediments. The Lp-PLA_2_ enzyme can be used as a nutritional intervention to lower the chances of developing cardiovascular diseases (CVDs) [10–12].

Certain strains of probiotics have been shown to be effective in controlling blood sugar levels and lowering weight and insulin peak levels in diabetic patients. Additionally, probiotics were found to be effective in relieving chronic inflammation which may lead to a reduced risk of developing cardiovascular diseases in these patients [13, 14]. The composition of gut microbiota differs between T2D and non-diabetic individuals, with increased risks of low-grade chronic inflammation in patients with T2D. Unhealthy gut microflora could therefore be linked to gut barrier disorders [15, 16]. Nonetheless, a number of studies have demonstrated a correlation between the level of Lp-PLA2 and inflammatory markers [17, 18], and between probiotics and type 2 diabetes [19, 20]. However, there are insufficient research regarding the amount of Lp-PLA2 in patients with T2D [9, 13, 15, 16]. In our previous research on patients with T2D, we investigated the effects of alpha lipoic acid, as an antioxidant with potential cardioprotective properties, on the Lp-PLA2 mass. We found that ALA may decrease the CVD risk by reducing Lp-PLA2 mass and improving the Lp-PLA2 distribution among lipoproteins in patients with T2D. Overall, in the T2D population, the effect of probiotics on this enzyme is equivocal [21].

The purpose of this randomized, double-blind, placebo-controlled clinical trial was to examine the effect of multi-strain probiotic supplementation on Lp-PLA2 as the primary biomarker, as well as glycemic parameters, lipid profile, body composition, and anthropometrics in patients with T2D.

## Methods and materials

### Design and participants

This 12-week randomized, double-blind, placebo-controlled clinical trial was conducted at Diabetes and Endocrine diseases Hospital in Sulaymaniyah, Iraq. The number of participants was 68 adult males and females, who were diagnosed as T2D for at least two years. The protocol was approved by both Ethics Committee of Tehran University of Medical Sciences (NO. IR.TUMS.MEDCINE.REC.1400.702) and Research Ethics Committee in Sulaymaniyah general directorate of health. In accordance to the contents of the 1975 Declaration of Helsinki as revised in 1983 and was registered at the Iranian Registry of Clinical Trials (IRCTID: IRCT20210529051435N1). Sample size was defined based on primary information in previous studies [22]. Glycemic and lipid profile variables were used to define the sample size, individually and finally total cholesterol (TC) level was found to be as the key variable which resulted in a maximum determined sample size [22, 23]. The findings from previous trials suggested plasma Lp-PLA2 mass to be positively associated with TC [24]. For an expected change of 0.25 mmol/L (9.65 mg/dL) between intervention and control groups and by considering α = 0.05 and a power of 80%, the sample size was computed to be 29.9 (≈ 30) per group which was increased to 34 to accommodate the expected dropout rate.

$$N = \left[ {{{(S1 + S2)}^2}{{({Z_{\frac{\alpha }{2}}} + {Z_\beta })}^2}} \right]/{\Delta ^2}$$

Eligible participants include those with established T2D for at least 2 years prior to the commencement of the study, aged 50–65 years, BMI between 25 and 34.9 kg / m^2^.

Exclusion criteria were, type 1 diabetes or non-diabetic patients, individuals who had irregular diet and unstable body weight (body-weight change > 5% within 3 months before screening), changing and/or addition in their routine medications within 3 months prior and during the intervention, history of cardiovascular disease, hypertension, heart attack, angina pectoris, cerebrovascular disease, stroke, Thyroid disease, and other chronic diseases and transmitted diseases in the past year. Current smokers, menopause, individuals on non-steroidal anti-inflammatory drugs and multivitamin or use of any nutritional supplements within the previous 3 weeks prior to study initiation, as well as the presence of liver, kidney, inflammatory or immunodeficiency diseases; thyroid disorders, use of any type of estrogen, progesterone, or diuretics; pregnancy or breast-feeding, and consumption of any type of probiotic product and/or antibiotic in the previous 2 months of testing. Participants who agreed to participate in the study were asked to sign a permission form after the lead researcher described the study protocol.

### Intervention

68 individuals were randomly assigned to two groups using a stratified block randomization approach and the principal researchers and all participants were blinded to the contents of the capsules throughout the study procedure and the final analysis. Participants were asked to receive either multispecies probiotic supplements (n = 34, i.e., 12 males and 22 females) or the placebo (n = 34, i.e., 8 males and 26 females). Supplements were either placebo (Placebo; containing starch and inactive ingredient excipients) or probiotics with dosages of Bacillus Coagulans, Lactobacillus plantarum, Lactobacillus acidophilus and Bifdobacterium bifdum, 3 billions/capsules (3 × 10^9^ colony forming units (cfu) per capsule). Oral capsules of Biodiab containing the mentioned strains which was produced by Tak-Gene Zist pharmaceutical manufacturer were taken by participants. Both probiotic and placebo capsules were produced and coded by the same manufacturer company (A or B). The packaging, odor, and appearance of placebo containing capsules were just similar to the supplement containing capsules. Each capsule was swallowed orally with a full glass of water. Participants from each group took one Biodiab capsules daily. A full pack of capsule was given to each participant monthly, then at any visits (biweekly) we counted the capsules to be sure about participant compliance, and if at the end a participant did not take ≥ 80% of the supplement we would exclude it from our results. A general information sheet was completed for each patient.

### Anthropometric measurements, dietary intake and physical activity

Body weight was measured by a calibrated scale with 0.1 kg accuracy (Seca, Hamburg, Germany) with barefoot and minimal clothes. Height was measured by a wall-mounted tape, barefoot and at a straight standing posture with 0.5-centimeter accuracy. BMI was calculated by dividing weight in kilograms by height in meters squared. Waist circumference (directly on the skin) was measured at the umbilical level after normal expiration with the subject in an upright standing posture using a plastic measuring tape with measurements to the nearest 0.1 cm. Anthropometric parameters was assessed at screening, baseline and 12th week. Body composition and fluid status was assessed by bioimpedance analysis (BIA) using the Inbody 770 (InBody co, Seoul, Korea). Bioimpedance measurements performed at a spectrum of 50 frequencies between 5 and 1000 kHz enable to differentiate between intra- and extracellular fluid, as low electronic currents cannot pass cell membranes and flow through extracellular water only. We recorded the full report of BIA while we analyzed fat mass (FM) and fat free mass (FFM).

To assess participant’s food intake, we collected 24 h food recalls at the baseline and end of the study. A trained nutritionist asked the patients to recall their intake by using a specific set of questions to gain as much detailed information as possible. To take the best result, for better estimating the portion size of foods and drinks, we used the Household Measures and Food Model booklet. Dietary intake was analyzed in terms of energy and macro - micronutrients (with a focus on micronutrients with fermented food) intake by Nutritionist software version IV.

To evaluate the physical activity levels, the short form of International Physical Activity Questionnaires (IPAQ) was used at the baseline and the end of the study. The short form of IPAQ includes seven questions that explore walking and moderate-intensity and vigorous-intensity activity during the past seven days. Frequency (days per week) and duration (time per day) have been collected separately for each specific type of activity. To analyze the activities, metabolic equivalents per minute (MET-minute) score was used. The MET-minute score was computed by multiplying the MET score by the minutes performed and used categorical score. The patients with at least 3000 met/minute score was obtained from the combination of all the activities were considered category 3 (high) and with at least 600 met/minute score were considered category 2 (moderate), and patients not included in category 2 or 3 were considered category 1 (low) [25].

### Laboratory measurements

Venous blood samples (10 ml) were drawn by venipuncture after 8 to 12 h fasting at baseline and the end of the study. The blood samples were centrifuged for 10 min at 3000 rpm using (Hettich centrifuge “Germany”), and serum will aliquot into separate micro tubes and were stored in -80 °C until biochemical analyses.

Serum levels of glucose, insulin, total cholesterol (TC), low density lipoprotein (LDL), high density lipoprotein (HDL), triglyceride (TG) and blood level of glycated hemoglobin (HbA1c) were measured at once and at the same place using an auto analyzer (Cobass 6000 Roche “Germany”). Insulin resistance was defined by calculating the HOMA-IR using Matthews et al.’s equation [26]: *fasting glucose concentration (mg/dl) × fasting insulin level (mU/l)/405*.

Lp-PLA2 serum level was measured by using an ELISA kit (diaDexus, Inc. South San Francisco, CA, USA).

### Statistical analysis

The obtained data was analyzed with SPSS, version 25 (SPSS Inc. Chicago, IL, USA). An Intention-To-Treat (ITT) approach was used for data analysis. In this approach, all the enrolled participants who take ≥ one capsule were included. Results were expressed as mean ± standard deviation (SD) and percentage for quantitative and qualitative variables, respectively. At the baseline, quantitative variables were analyzed by independent sample t-test. Chi square test was used to compare qualitative variables. The repeated measure analysis of variance (ANOVA) was used to report the results after correcting for sex, total energy intake and body fat mass. A P value equal and less than 0.05 was considered statistically significant.

## Results

### Recruitment and subject flow

**Fig. 1:**
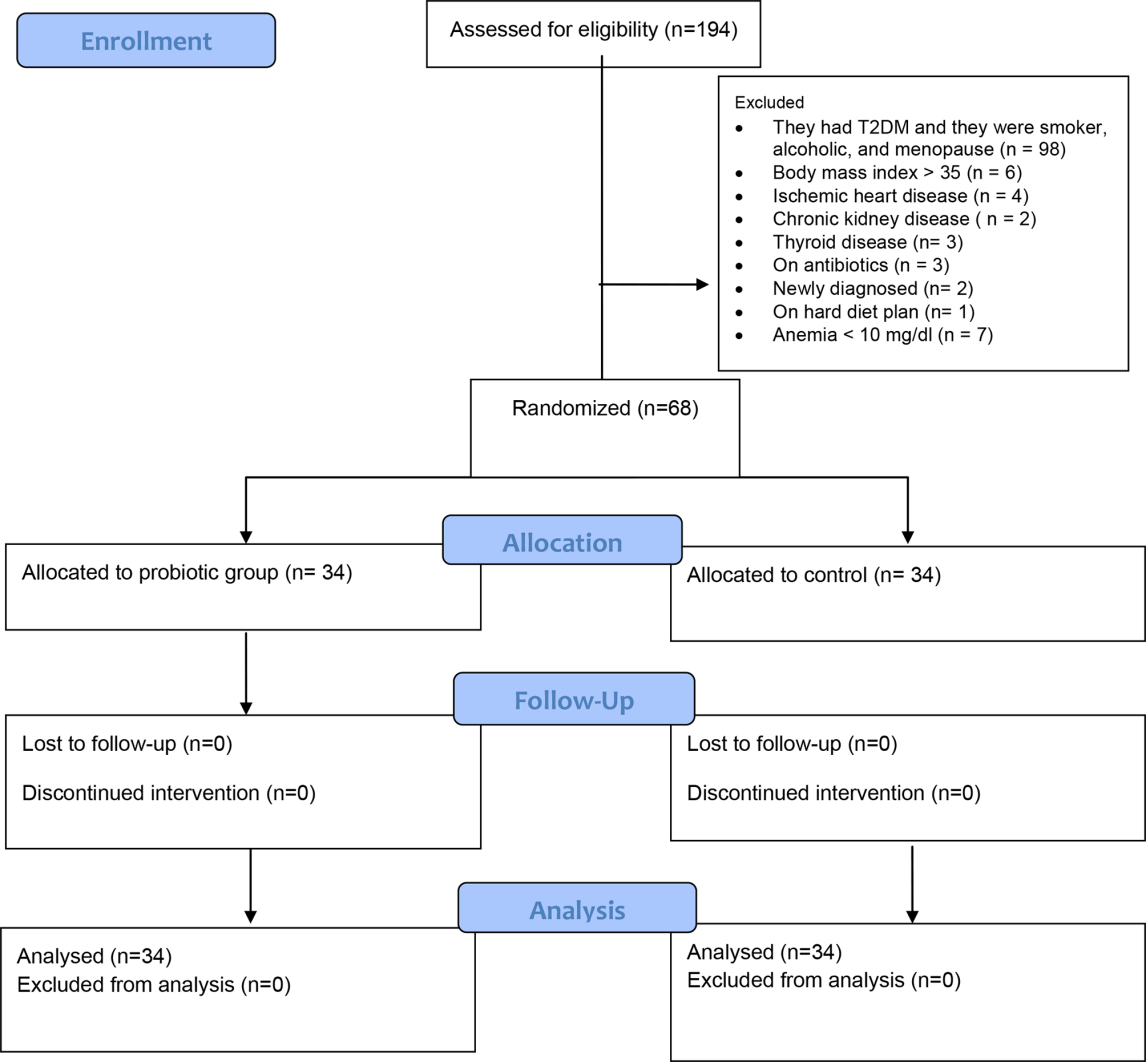
Diagram of the study design

A total of 194 patients were studied, of whom 68 were identified as eligible and have an updated contact in the hospital system. 34 participants in the probiotics group and 34 participants in the placebo group successfully completed the intervention with a compliance rate of > 85% (Fig. 1). All of the participants routinely consumed probiotic supplements or food sources rich in probiotics. The supplement capsule was generally acceptable to participants, and they were satisfied with the taste and smell of it. All the participants who assigned the consent were loyal to our protocol and they completed intervention (Table 1).

**Table 1:** Baseline characteristics of the study participants

	Probiotic (n = 34) Mean±*SD*	Placebo (n = 34) Mean±*SD*	*P* Value
Age (year)	55.6 ± 4.7	56.5 ± 4.8	0.4
Sex, n (%)			0.3
male	11 (32%)	8 (24%)	
female	23 (68%)	26 (76%)	
T2D duration (year)	8.03±4.3	7.94±5.3	0.7
Anthropometrics			
Weight (kg)	81±11.06	77.9±11.4	0.35
BMI (kg/m^2^)	31.2±3.3	31.1±3.4	0.6
WC (cm)	105.7±8.2	105.8±9.6	0.95
FM (kg)	28.2±7	28.9±6.6	0.86
FFM (kg)	52±9.8	49.07±8.1	0.08
Physical activity level			
High, n (%)	2 (5.8%)	1 (3%)	0.16
Moderate, n (%)	12(35.2%)	6 (17.6%)	
Low, n (%)	20 (58.8%)	27 (79.3%)	

BMI body mass index, FM; fat mass, FFM; fat free mass, FBS fasting blood sugar, HbA1c glycated hemoglobin, HOMA-IR homeostasis model assessment-estimated insulin resistance, TC total cholesterol, TG: Triglyceride, HDL: High Density of Lipoprotein, LDL: Low Density of Lipoprotein, Lp-PLA2 lipoprotein phospholipase A2, * Significant difference (p < 0.05). Data are presented as Mean ±SD and Frequency (Percent). Student t-test and chi-square test were used to compare quantitative and qualitative variables, respectively

### Demographic characteristics

A total of 68 participants (29.4% male and 70.6% female) were allocated to the probiotics (n = 34) or placebo (n = 34) group. The mean ages of the probiotics and placebo group were 55.6 and 56.5 years, respectively. Table 2 shows the baseline characteristics of participants. There were no significant differences in age, gender, and T2D duration between the two groups at baseline. None of them were on a certain diet. Mean of body weight, waist circumference, body mass index (BMI), FM and FFM were also non-significant between two groups at baseline.

**Table 2:** Dietary intake throughout the study in probiotics and placebo group

	Probiotic (n = 34)			Placebo (n = 34)			P values		
	Baseline	After	Change	Baseline	After	Change	Time	Group	Tim * Group
Energy (kcal/day)	2553±368	2580±360	-27	2777±274	2741±260	36	0.24	0.008	0.066
Protein (g/day)	100±19	98±15	2	111±15	113±14	-2	0.16	0.001	0.08
Carbohydrate (g/day)	395±80	381±67	14	396±63	384±63	12	0.06	0.8	0.1
Fat (g/day)	68±21	80±10	-12	90±14	90±15	0	0.73	< 0.001	0.004*

Data are presented as Mean ±SD, Statistic tests: repeated measure analysis of variance

* Significant difference (p < 0.05)

### Changes in dietary intake and physical activity level

Physical activity levels did not differ between two groups. The change of energy intake between two groups (time*group) was not significantly different. Similarly, the mean change of protein and carbohydrate between two groups was not significant. In contrast, patients after intake of probiotic had a higher fat intake compared to placebo (68 ± 21 in the probiotic group and 90 ± 14 in the placebo group at baseline vs. 80 ± 10 in the probiotic group and 90 ± 15 in the placebo group at week 12 (p < 0.001), (Table 2).

### Changes in primary outcome

The serum level of Lp-PLA2 was significantly decreased in probiotic group (47.4 ± 7.5 at baseline and 41 ± 7 after 12 weeks) compared to placebo group (p = < 0.001) (Table 3).

**Table 3:** Effects of probiotic supplementation on anthropometric measures, body composition and laboratory measurements

Variables	Probiotic (n = 34)			Placebo (n = 34)			P values		
	Baseline	After	Change	Baseline	After	Change	Time	Group	Time * Group
Weight (kg)	81±11.06	81.5±12	0.5	77.9±11.4	77.7±11.2	0.2	0.94	0.35	0.72
BMI (kg/m^2^)	31.2±3.3	30.4±6.3	0.8	31.1±3.4	31.01±3.5	0.09	0.96	0.6	0.11
WC (cm)	105.7±8.2	101.4±19.3	4.3	105.8±9.6	105.56±9.5	0.24	0.65	0.95	0.2
FM (kg)	28.2±7	27.3±8	0.9	28.9±6.6	28.87±6.7	0.03	0.04	0.86	0.42
FFM (kg)	52±9.8	53.7±11	1.7	49.07±8.1	48.8±7.7	0.27	0.9	0.08	0.07
FBS (mg/dl)	207±74	176±53	31	184.6±57	176.5±52	8.1	0.44	0.37	0.06
HbA1c (%)	9.9±2.4	8.4±1.4	1.5	8.36±1.6	8.33±1.3	0.03	0.98	0.09	< 0.001*
Insulin(µU/mL)	16.9±13.4	15.3±7.4	1.6	22.27±19.6	23.1±21	-0.83	0.56	0.22	0.28
HOMA-IR	7.85±5.3	6.7±5.1	1.15	10.48±9.6	10.49±10.3	-0.01	0.3	0.21	0.26
TC(mg/dl)	186±50	181±40	5.586	168±36	169±28	-1.65	0.37	0.06	0.42
LDL(mg/dl)	131.75±43	117±37	14.694	108.15±38	107.47±30	0.68	0.6	0.06	0.08
HDL(mg/dl)	38±8	44±8.5	-6	40.3±8.8	41.6±9.2	-1.3	0.87	0.78	0.005*
TG(mg/dl)	152.3±68	141.7±60	10.6	128.5±55	135.85±59	-7.35	0.76	0.1	0.16
Lp-PLA2(ng/ml)	47.4±7.5	41±7	6.4	46.13±7.1	47.3±7.5	-1.17	0.3	0.16	< 0.001*

BMI body mass index, FM; fat mass, FFM; fat free mass, FBS fasting blood sugar, HbA1c glycated hemoglobin, HOMA-IR homeostasis model assessment-estimated insulin resistance, TC total cholesterol, TG: Triglyceride, HDL: High Density of Lipoprotein, LDL: Low Density of Lipoprotein, Lp-PLA2 lipoprotein phospholipase A2, * Significant difference (p < 0.05). Data are presented as Mean ±SD, Statistic test; repeated measure analysis of variance

### Change in secondary outcomes

Repeated measure ANOVA showed that HbA1c was not significantly different between both groups at baseline, while after intervention, a significant decrease was observed after 12 weeks in the probiotic group by 1.5% compared to placebo (p < 0.001) (Table 3). We did not observe any significant change in fasting blood sugar serum insulin, HOMA-IR, TC, TG, LDL anthropometric parameters (weight, WC, BMI), FM, and FFM within and between groups (Table 3).

The HDL levels improved in probiotic group comparing to placebo group (At baseline it was 38 ± 8 in probiotic group and 40.3 ± 8.8 in placebo group, while at the week 12 it was 44 ± 8.5 and 41.6 ± 9.2, respectively (p = 0.005) (Table 3).

## Discussion

This study revealed that probiotic supplementation effectively reduced Lp-PlA2 and HbA1c levels in T2D patients while increasing HDL levels. However, it has no significant effect on FBS, insulin resistance, weight, waist circumference, BMI, FM, FFM, TC, TG, or LDL profile.

Positive effect of probiotic in improvement of Lp-PLA2 was demonstrated in non-diabetic subjects, in this study was consistent with earlier research [27]. In the present study there was a significant reduction of serum level of Lp-PLA2 in probiotic group while the level of the enzyme in placebo group was not lowered; even it was raised a little. This study did not succeed to suggest a clear mechanism for the reducing effect of probiotic on Lp-PLA2 among T2D patients.

According to a recent study, the pro- or anti-inflammatory effects of Lp-PLA2 depend on how it was distributed relative to HDL and LDL. This concept states that whereas Lp-PLA2 was pro-inflammatory when attached to LDL, it was anti-inflammatory when bound to HDL [28]. In addition several findings have shown that Lp-PLA2 levels in diabetic patients are higher than in healthy individuals [9]. Furthermore, diabetic patients seemed to have a higher level of LDL bound Lp-PLA2 [29]. Studies have demonstrated elevated levels of Lp-PLA2 mass and activity in individuals with T2D compared to those without diabetes. Additionally, a strong correlation was found between high Lp-PLA2 levels and poor diabetes management [30–32]. It was an inherent risk factor for cardiovascular disease and inflammatory variables [33]. A meta-analysis of above 79,000 subjects discovered that increased Lp-PLA2 mass or activity raises the risk of coronary heart disease and stroke [34]. A former study had demonstrated the significant reduction in Lp-PLA2 level after probiotic supplementation for 12 weeks in non-diabetic subject [27]. Previous research has shown that inflammation and oxidative stress are major mechanisms for the progression of diabetes complications [35]. Probiotic interventions have been shown to inhibit and reduce pro-inflammatory factors like TNF-a, IL-6, and IL19 as well as increase antioxidant biomarkers like glutathione peroxidase and superoxide dismutase [36]. Hence, it was possible to conclude that probiotic supplementation in diabetic individuals must be beneficial in improving Lp-PLA2 enzyme. Importantly this was a strong excuse for positive effects of probiotics on T2D.

There was improvement of glycemic parameters in the present study like the previous studies [37–39]. Nevertheless, some trials found no glycemic control improvements with probiotic supplementation [40–42]. Mazloom et al. reported lack of beneficial effects of probiotics on inflammatory biomarkers which could be attributed to the limited sample size [42]. The medications used by the patients may also contribute to the disparity in results between researches. In terms of medications, different studies enforced varied inclusion criteria. Probiotics may have influenced pharmacodynamics in animal model [43].

Probiotics supplementation in our study significantly reduced HbA1c, whereas there was no significant influence on FBS. Actually, several factors, such as the time have passed since the previous meal [44], physical activity [45], and a variety of other neurology and endocrinology parameters [46], have an impact on FBS. Thus, it was challenging to determine any change in the FBS levels as a result of intervention.

We did not observe significant changes in other glycemic controls like insulin and HOMA-IR. A recent trial revealed that administration of two strains of probiotic supplements (Lactobacillus and Bifidobacterium) resulted in no significant changes in FBS and lipid profiles [39]. Although they reported Probiotics improved HbA1c and fasting insulin in people with type 2 diabetes, the reduction in HOMA-IR in intervention group compared to placebo was not significant [39].

In our study any significant change in weight, BMI, and body composition (FM and FFM) did not observe within and between groups. Generally, people who have diabetes have a harder time losing weight than healthy persons, especially when the weight loss combines with an improvement in glycemic control [39]. Improvements in glycemic control will also result in decreased energy expenditure in individuals with poorly controlled diabetes, which inhibits additional weight loss [39].

Similarly, in lipid profile measurements there was no significant change in TC, TG and LDL within and between groups throughout the study. Inconvenient data have been published on the impact of probiotics on lipid profiles; similar to our findings, a prior research revealed no significant changes in the levels of TC [47]. However, another research using fermented milk in the probiotic group found a substantial reduction in TC and LDL levels in between group comparisons, which was not significant in within group analyses [48]. Furthermore, contrary to our findings, a research found a substantial decrease in HDL levels when receiving probiotic capsules [38]. Likewise, a couple of studies revealed a substantial rise in HDL levels [23] following 8 weeks of intervention with probiotic yogurt [49]. Because HDL transports cholesterol in the form of cholesteryl esters to the liver for further hydrolysis, it has been postulated that probiotics or synbiotics might generate a hypocholesterolemic impact by modifying the pathways of cholesteryl esters and lipoprotein transporters [50]. Probiotics’ lipid-lowering mechanisms have been found to involve enzymatic deconjugation of bile acids, ingestion of cholesterol by probiotic cell membranes, and, more substantially, the formation of short chain fatty acids by probiotics during fermentation, which might reduce cholesterol synthesis [23].

The study’s limitations should be addressed. The main researcher was in charge of randomization, although it would have been preferable if the randomization sequence had been revealed by a third party. Another limitation was the participants’ lower educational level; they might modify their usual diet without informing the researcher. Moreover, the short duration of the study may also be another reason that diabetes problems often develop gradually over time. Although we used 24-h food recall which is a commonly used dietary assessment method in food consumption, food record (2 weekdays and 1 weekend day) might be more accurate than recall. A 12-week intervention might not be sufficient to reverse the complications. Additionally, dietary recommendations to both groups could help the participants do not change their diet.

## Conclusion

We found that multi-strain probiotic supplementation in patients with type 2 diabetes for 12 weeks may improve Lp-PLA2, HbA1c and HDL levels. However, more research is needed to support our results and establish probable underlying processes.

## Data Availability

Authors declare that all relevant data are included in the article and/or its supplementary information files will be available upon request.
